# Intrathecal Baclofen Therapy in Russia: National Register, the Effect of One-Year Usage

**DOI:** 10.17691/stm2020.12.1.10

**Published:** 2020

**Authors:** E.V. Filatov, A.R. Biktimirov, A.I. Simatov, A.A. Tomsky, A.I. Ushakov, A.G. Timershin, D.V. Krestchenok, M.S. Levin, K.V. Nesterin, O.N. Kirsanova, E.O. Posidelova, A.V. Vtorov, M.N. Klochkov, A.E. Polyakov, K.V. Semkin, E.R. Siid Abla, I.N. Morozov

**Affiliations:** Head of Neurosurgery Department, Novokuznetsk Scientific and Practical Centre of Medical and Social Assessment and Rehabilitation of Disabled, Ministry of Labor and Social Protection of the Russian Federation, 7 Malaya St., Novokuznetsk, 654055, Russia; Neurosurgeon, Neurosurgery Department, Medical Center of Far Eastern Federal University, 10/25 Village Ayaks, Island Russkiy, Vladivostok, 690922, Russia; Director, LLC “Envil-Soft”, 115 Akademika Sakharova St., Nizhny Novgorod, 603136, Russia; Head of Functional Neurosurgery Group, N.N. Burdenko National Medical Research Center for Neurosurgery, Ministry of Health of the Russian Federation, 16, 4-ya Tverskaya-Yamskaya St., Moscow, 125047, Russia; Neurosurgeon, University Clinic, Privolzhsky Research Medical University, 10/1 Minin and Pozharsky Square, Nizhny Novgorod, Russia; Head of Neurosurgery Department, Republican Children Clinical Hospital, 98 Stepana Kuvykina St., Ufa, Bashkortostan, 450106, Russia; Neurosurgeon, Neurosurgery Department, Republican Children Clinical Hospital, 98 Stepana Kuvykina St., Ufa, Bashkortostan, 450106, Russia; Neurosurgeon, Republican Clinical Hospital, 138 Orenburgskiy trakt St., Kazan, Republic of Tatarstan, 420064, Russia; Head of Neurology Department, City Clinical Hospital No.1, 46 Traktorostroiteley Avenue, Cheboksary, Chuvash Republic, 428028, Russia; Neurosurgeon, P.A. Gertsen Moscow Research Oncology Institute — Branch of the National Medical Research Radiological Center, Ministry of Health of the Russian Federation, 3, 2-y Botkinskiy Proezd, Moscow, 125284, Russia; Neurologist, Almazov National Medical Research Center, Ministry of Health of the Russian Federation, 2 Akkuratova St., Saint Petersburg, 197341, Russia; Head of Neurosurgery Department, V.M. Bekhterev National Medical Research Center for Psychiatry and Neurology, Ministry of Health of the Russian Federation, 3 Bekhtereva St., Saint Petersburg, 192019, Russia; Neurosurgeon, Neurosurgery Department, V.M. Bekhterev National Medical Research Center for Psychiatry and Neurology, Ministry of Health of the Russian Federation, 3 Bekhtereva St., Saint Petersburg, 192019, Russia; Neurosurgeon, Neurosurgery Department, Territorial Clinical Hospital, 3а Partizana Zheleznyaka St., Krasnoyarsk, 660022, Russia; Head of Neurosurgery Department, Simferopol Clinical Emergency Care Hospital No.6, 15 Gagarina St., Simferopol, Republic of Crimea, 295026, Russia; Neurosurgeon, Neurosurgery Department, Simferopol Clinical Emergency Care Hospital No.6, 15 Gagarina St., Simferopol, Republic of Crimea, 295026, Russia; Head of Center of Neurorehabilitation of Patients with Spine Cord Pathology, Privolzhsky Research Medical University, 10/1 Minin and Pozharsky Square, Nizhny Novgorod, Russia

**Keywords:** severe muscular spasticity, intrathecal baclofen therapy, treatment register of spastic states

## Abstract

Patients with severe muscular spasticity still represent the most complex and resistant to therapy group of neuro-rehabilitation patients. In a few years, in Russia, intrathecal baclofen therapy has appeared to be the most effective method for such spasticity. For the first time the authors developed and implemented in clinical practice “Prospective register to treat spastic states using intrathecal baclofen therapy in Russian Federation” aimed at therapy classification of spastic patients: to reveal management characteristics, assess treatment outcomes and frequency of occurrence of adverse events that will finally help specify the need for the method employment in real clinical practice.

The article presents the findings of a one-year usage of Register, which enabled to make a preliminary evaluation of intrathecal baclofen therapy in Russia.

## Introduction

Spasticity, as one of the constituents of upper neuron syndrome, is characterized by muscle tone alterations dependent on spread rate, and recurrent involuntary skeletal muscle contractions (spasms) [1]. Muscular rigidity hinders patient’s movements, involuntary jerks of the limb and the whole body affect patient’s sleep, mobility and self-care ability. Disabling high muscle tone is found in 40–68% of patients after spinal and cerebrospinal injuries, and in 65–84% of patients with multiple sclerosis [1]. Severe muscular spasticity occurs in 20–25% of post-stroke patients [2–6], and the incidence of severe muscular spasticity after severe craniocerebral trauma reaches 85% [7–10]. Muscle hypertonia becomes an urgent issue in children. Spastic pareses develop in 65–98% of children with cerebral palsy [11–13]. Pathologically changed muscular tone in patients after myelitis, spinal stroke and myeloischemia is less studied.

Conventional treatment of spasticity suggests therapeutic exercises, massage, reflexotherapy, physiotherapy, administration of botulinal toxins in motor points of spastically contracted muscles. Botulin-therapy is the most effective in equinovalgus caused by the spasticity of calf muscles, or in case of high muscle tone of wrist flexor and flexor digitorum, i.e. in localized, focal spasticity [2, 14, 15]. A surgery to reduce spasticity is possible at the following four levels — brain (electrocoagulation of pallidum, ventral lateral nucleus of thalamus or cerebellum), spinal marrow (selective posterior rhizotomy), peripheral nerves (peripheral neurotomy) and muscles and their tendons.

Before the 80s of the XX century, destructive neurosurgical procedures: posterior selective rhizotomy and DREZ-tomy were the main techniques to treat severe spastic syndromes. Their effect was based on destructing a stretch-reflex chain that enabled reflectory spasticity decrease. Despite their high efficiency, the techniques have one essential fault: in some cases uncontrolled muscular weakness can develop in postoperative patients, and it disturbs locomotor functions in those patients who use muscular tone when walking.

Baclofen has become a widely applied medication for spasticity therapy since 1971 when it was introduced in medical practice [16]. However, when it is taken orally, it hardly crosses blood-brain barrier, therefore, to have a therapeutic effect, it is necessary to achieve an appropriate Baclofen level in cerebrospinal fluid, and its high concentration in blood as well. The administration of large therapeutic dosages of oral neuromuscular relaxants is likely to result in side effects [8, 9]. The first intrathecal baclofen administration was reported in 1984, its therapeutic concentration in cerebrospinal fluid was achieved by the dosage 400–1000 times as less than that when taken orally. Since that time intrathecal baclofen (ITB) therapy using implantable pumps is an effective method to treat nonfocal spastic syndromes: since 1993 — infantile cerebral paralysis (ICP), and since 2000 — hyperkineses and secondary dystonia of different etiology making worse patients’ life quality, as well as resistant to conventional surgical and drug therapy [1, 3, 5, 17, 18]. Intrathecal baclofen therapy has been used in Russia since 2010 (the first baclofen pump was implanted in Russia in 2000).

In USA, in 2016, there were registered 649 thousand patients with spasticity due to ICP, and 268 thousand patients — due to multiple sclerosis, the drug-resistant forms being revealed in 50 and 38% of cases, respectively; in 172 thousand patients spasticity developed due to spinal and spine cord injuries, and in 1.5 million patients — after cerebral stroke. Disabling muscular tone in 83 thousand spinal patients and in 438 thousand post-stroke patients was resistant to drug therapy, kinesitherapy and physiotherapy [19, 20]. Over 280,000 pumps have been implanted for 30 years, since the time intrathecal baclofen therapy was introduced.

Currently, in Russia there are the data on over 100,000 patients with muscular spasticity due to spinal injuries, the muscles of one third of the patients are resistant to conventional therapy [21, 22].

Annually, over 400,000 strokes are registered in Russia, spastic hemiparesis (up to 80% of cases) being the main disabling post-stroke defect. In Russia there are no reliable data on drug-resistant spasticity deteriorating life quality of patients with demyelinating diseases, ICP, cerebral pathology. However, considering a large population of such patients, one may state that objective demand for intrathecal baclofen therapy in Russia is much higher than the number of implanted baclofen pumps: within 7 years, while the method has been used in Russia, a little more than 500 pumps were implanted.

To develop intrathecal baclofen therapy in Russia as a part of high-tech medical care, it is necessary to develop an adequate patient selection system, and follow the requirements for the method application, correctly adjust a daily dosage and dose schedule, to organize a competent long-term follow-up and education of patients after pump implantation, as well as there should be reliable data on using ITB therapy in Russian patients according to the adopted indications, its efficiency and safety [1, 23].

“Prospective register to treat spastic states using intrathecal baclofen therapy in Russian Federation” was developed and introduced in January 2019. The Register objects are: to acquire the data from Russian centers using intrathecal baclofen therapy in patients with spastic conditions; data classification, analysis, evaluation of treatment outcomes and adverse events with subsequent practical guidelines [24].

**The aim of the present publication** was to consider the first results of the Register usage in Russia.

## Materials and Methods

According to the Register, intrathecal baclofen therapy state in Russia appears to have the following characteristics:

previous neuromuscular relaxant therapy in patients with implantation;

indications for pump implantation including the compliance of the provided treatment with the indications;

refilling time, visit frequency for pump programming;

pump programming parameters.

To assess ITB therapy the following scales were used:

muscular spasticity (according to Ashworth scale);

muscle spasm rate (homonymous scale);

the intensity of a pain syndrome due to spasticity (according to VAS);

disability (according to a modified Rankin disability scale);

patient’s life quality (according to Karnofsky performance status).

To assess the therapy safety we systemized adverse events due to baclofen pharmacokinetics and pharmacodynamics, as well as those associated with the procedure and/or the device. The data enabled to establish the reason and frequency of baclofen therapy complications.

In Russia, ITB therapy patients are watched in 26 centers including 7 centers where children are managed. The Register includes all patients (adults and children) with implanted pump for ITB therapy regardless the prescription of the therapy used (at the stage of pump implantation, pump re-implantation after the termination of its lifetime, pump refilling and/or reprogramming) and the differences in indications meeting the inclusion criteria.

Inclusion criteria were the following:

1. a signed informed consent to be included in Register (children needed two consents: one from a child and another from parents/legal representatives);
2. an implanted pump for intrathecal baclofen therapy.

A non-inclusion criterion for Register was a patient’s refusal to sign an informed consent.

Exclusion criteria were:

1. a voluntary refusal of a patient to be included in Register;
2. the necessity for pump explantation;
3. patient’s death.

To enter in Register, there was developed a protocol of “Prospective register to treat spastic states using intrathecal baclofen therapy in Russian Federation” to describe every visit, with assessment tools and automated information data system given available at special websites via the employees of research centers. An electronic case report form was developed, which was completed during patients’ visits (to enter in Register, when being followed up, and during a final visit); it enabled to gain the information on demographic (registration) and clinical laboratory data; therapy efficiency (assessment scales for spasticity, pain, the quality of life and activity); pump working conditions and adverse events.

## Results

Over the year 2019, 160 people from 18 centers entered the Register, it amounting 35.5% of all patients receiving ITB therapy in the Russian Federation. 89% of all patients were registered in 7 centers (Moscow, Novokuznetsk, Saint Petersburg, Nizhny Novgorod, Ufa, Kazan, island Russkiy). Three centers had two patients, and other three centers had one patient (the centers are regional and territorial hospitals, their patients had the pumps implanted in central clinics of the Russian Federation). Among all the patients studied, 70.1% are men/boys, 29.9% — women/girls, 32% — children. Patients’ mean age was 31.5 years old.

A median of neuromuscular relaxant intake time before pump implantation was 57.5 [37.7; 96.0] months. A minimal baclofen intake period before intrathecal infusion was 3 months. Most frequently (in 92.9% of all cases with side effects), the side effects of oral administration of neuromuscular relaxants were: dizziness — in 25.9% of cases, constipations — in 18.8%, sleep disturbance — in 17.6%, muscle weakness — in 16.5%, and arterial hypotension — in 14.1%.

Spasticity causes:

spine cord injury — 29.3%;

ICP — 38.4%;

myelitis and other spinal inflammatory diseases — 5.5%;

spinal stroke — 4.9%;

multiple sclerosis — 9.1%;

craniocerebral trauma — 4.9%;

others — 7.9%.

Among the patients with ITB therapy prevailed those with spastic quadriparesis — 62.6%, spastic paraparesis was diagnosed in 31.6%, hemiparesis — in 4.5%, and triparesis — in 1.3%.

As of the date of Register entry, maximum high muscle tone of lower limbs (score 5) was diagnosed in 6.6% of patients, among ICP patients there were 8.6% of such patients, among the patients after spinal injury — 5.5%. High tone (score 4) was revealed in 20% of studied patients: in 20.7% of ICP patients and in 62% after severe craniocerebral trauma.

Muscular tone of lower limbs — score 3 as of the date of Register entry — was diagnosed in 30.4% of patients, and 25% were found among those with spine cord injuries, with ICP — 31.6%, with multiple sclerosis — 45.5%.

Increased muscle tone of upper limbs was revealed in 59% of patients, maximum muscle tone being found in patients after severe brain injury (score 4 [3; 5]). Maximum tone values (score 5) in arms were revealed in 6% of patients, score 4 — in 15%. More often the score was not higher than 3 — in 38% of patients under study, score 2 tone was found in 28% and score 1 — in 13%.

Muscular spasms were recorded in 43.8% of patients, in 2/3 of them the spasms resulted from spinal injury or ICP.

In muscular spasticity, pain syndrome of different intensity was diagnosed in 25% of examined patients; only adults and 1 child complained of pain. 76% of cases with pain were male patients. Among the patients with pain syndrome there prevailed those whose daily baclofen dosage was up to 130 μg (70%), in 30% a daily dosage was from 150 to 290 μg. The patients with 300 μg dosage per day felt no pain. All the patients who had impaired spinal circulation felt 30 mm [25; 35] pain according to VAS, maximum intensity being up to 90 mm.

Most ITB patients experienced major violations of life activities as evidenced by maximum scores according to modified Rankin disability scale (5 and 4) — in 17.6 and 61.5% of patients, respectively; the patients with craniocerebral trauma and those with ICP appeared to have maximum impaired self-care and mobility. Score 3: life quality deterioration was found in 15.7% of patients, score 2 — in 4.5%, and score 1 — in 1.3%.

Life quality assessment (Karnofsky scale) showed 6.7% of patients with ITB therapy to have no need for special care (index 80–100); 52.5% exhibited work inhibition with complete self-reliance (score 50–70); self-care inability when care and hospitalization are required (score 40 and lower) was revealed in 40.8% of patients. Best life quality characteristics were found in patients with multiple sclerosis (60 [60; 70]) and sequalae of impaired spinal circulation (70 [45; 70]).

The median of pump implantation time as of the time of Register entry was 37.7 [16; 55] months.

The analysis showed the most popular baclofen concentration in pump implantation to be 500 μg in 1 ml — in 70.1% of patients, and such concentration remained in most of them within the whole follow up period; maximum baclofen concentration (2000 μg/ml) was found in 17.6% of cases. In rest of the patients the concentration distribution was as follows: 250 μg/ml — in 1.3%; 750 — in 0.9%; 1000 — in 6.5%; 1250 — in 1.1%; 1500 — in 2.5% of patients.

The dose schedule at equal rate and, therefore, an unchanged dose within 24 h prevailed in 95.4% of patients. Only 4.6% used a flex mode enabling to change baclofen injection rate throughout the day depending on a muscular tone required, no significant difference in a dose schedule being revealed in patients of different age and different diagnoses.

A daily dose of intrathecal baclofen in spinal injury sequelae, after myelitis, impaired spinal circulation was the same: 100 μg/day, maximum dose being 770 μg/day in a patient with craniocerebral trauma sequelae, and 1450 μg/day in a patient with Strumpell disease. Patients with multiple sclerosis needed a minimal baclofen dose: 70 [52.5; 75.0] μg. In muscle spasticity due to craniocerebral trauma a daily baclofen dose was 140 [100; 250] μg, its maximum being 420 μg. ICP patients required a significant daily dose of intrathecal baclofen: 151.5 [80; 240] μg, its maximum being 450 μg.

Serious adverse events, three quarters of which referred to severe, were diagnosed in 6.2% of Register subjects, one patent appeared to have three adverse events. The adverse events related to the procedure or a device were the problems associated with catheters: impaired patency, breakage, impaired connection with a pump — 25% of all events; infected pump pocket/ pump bedsores — 33.3%; liquorrhea accompanied by a recurrent subcutaneous cyst — 8.3%; the over-dosage with a clinical picture of diplopia, dizziness, arterial blood pressure fall up to syncope state — 16.6% of all conditions. Pump failure occurred in 16.8% of cases.

## Conclusion

A one-year work of Register “Prospective register to treat spastic states using intrathecal baclofen therapy in Russian Federation” enabled to preliminarily assess ITВ therapy in Russia.

The Register provided an opportunity to reveal the main indications for pump implantation in Russia; spine cord injury (29.4% of all implantations) and infantile cerebral paralysis (38.4%) rank the leading position that confirms a worldwide tendency.

Patients with intrathecal baclofen therapy had marked impaired life activities and life quality, 62% of implanted patients suffering from severe spastic tetraparesis. Muscular spasms restricting life activities and accompanied by pain syndrome were recorded in 43.8% of patients that shows the necessity for their diagnostics and therapy, which are not routine so far.

The register showed the time median of the previous neuromuscular relaxant therapy in implanted patients to be about 5 years. It can be the evidence of sufficient efficiency of both: oral baclofen administration and a long way a patient has to work up to intrathecal administration.

The study revealed the most popular baclofen concentrations in pump, as well as dose schedules giving the evidence that most experts prefer use a routine dose schedule; it may be related infeasibility/ unwillingness of repeated patients’ visits.

Not high rate of adverse events revealed within a year suggests a high level of functional neurosurgery in Russia; however, further studies are needed to specify the patient selection criteria in order to reduce complication risk, the common complications are pump bedsores in spinal patients.

A longer follow up is necessary to solve the problem of baclofen dose adjustment in patients of different nosological entities, the issue of a long-term efficiency of intrathecal therapy remaining urgent as well.
